# Genotypic Diversity of Feline Bocavirus Identified in Cats from Italy

**DOI:** 10.3390/pathogens15101040

**Published:** 2026-10-01

**Authors:** Irene Mauri, Alessia Facchin, Angelica Stranieri, Giulio Cocciolo, Saverio Paltrinieri, Stefania Lauzi

**Affiliations:** 1 Department of Veterinary Medicine and Animal Sciences, University of Milan, Via dell’Università 6, 26900 Lodi, Italy; irene.mauri@unimi.it (I.M.); alessia.facchin@guest.unimi.it (A.F.); angelica.stranieri@unimi.it (A.S.); stefania.lauzi@unimi.it (S.L.); 2 MyLav-Laboratorio di Analisi Veterinarie s.r.l., Via Giuseppe Sirtori 9, 20017-Rho Milano, Italy; giuliococciolo@mylav.net

**Keywords:** emerging virus, feline, gastrointestinal disease

## Abstract

**Background:** Feline bocavirus (FBoV) is considered one of the emerging viral agents affecting cats. **Methods:** this observational study investigated FBoV occurrence by convenience sampling of 224 cats admitted to the Veterinary Teaching Hospital of Lodi, Italy, in 2020–2025 using PCR targeting the NP1 gene. Positive samples were phylogenetically analyzed. **Results:** FBoV positivity was observed in 17/224 (7.6%) cats. FBoV genotypes 1 (4%), 2 (0.9%) and 3 (2.7%) were identified. Out of the animals with collection of fecal samples, FBoV-positive cats were 17/53 (32.1%), whereas cats with blood (n. 69), respiratory (n. 54), urine (n. 46), or other (n. 2) samples were FBoV-negative. In a subset of cats with more than one fecal sample collected, viral carriage was detected for up to two weeks. Young age, stray/shelter condition and hospitalization were significantly associated with FBoV positivity (*p* = 0.0008, *p* = 0.0139 and *p* = 0.0023, respectively). In cats with analyzed fecal samples, Feline panleukopenia virus (FPV) positivity was associated with FBoV positivity (*p* < 0.001), whereas presence of diarrhea was not associated with FBoV occurrence. **Conclusions:** the sampling strategy did not allow us to estimate FBoV prevalence in the cat population. Nevertheless, the circulation of genetically diverse FBoV strains among cats in Italy, the association of FBoV positivity with hospitalization and results suggesting that FBoV may not be a primary pathogen for gastrointestinal disease underscore the importance of comprehensive surveillance studies to enhance the understanding of the evolutionary characteristics, epidemiology and pathogenic role of FBoV.

## 1. Introduction

Bocaparvoviruses (BoVs), formerly known as bocaviruses, are an emerging group of viruses in the family *Parvoviridae* that were first identified in 2005 in children affected by acute lower respiratory tract infections and gastroenteritis and were later detected in a variety of animal species, indicating a broad host range [1,2,3,4]. Transmission of BoVs commonly occurs via the fecal–oral route, leading to respiratory and gastrointestinal signs in young animals and humans, whereas infections in adults are frequently subclinical [5,6,7]. Human bocavirus (HBoV) has also been occasionally detected in serum and urine of infected children, suggesting the presence of other possible HBoV-associated disease [8,9].

Feline bocavirus (FBoV) was first identified in 2012 in fecal, urine, kidney, nasal and blood samples from stray cats with or without clinical signs in Hong Kong [10] and is now considered one of the emerging viral agents affecting cats [4,11,12]. To date, besides other studies detecting FBoVs in cats from China [2,3,13,14], FBoVs have been reported in several countries, including the USA [15], Canada [11], Japan [16], Thailand [17,18], Vietnam [19], Belgium [20], Portugal [21], Turkey [22] and Southern Italy [23].

Three genotypes of FBoV (FBoV-1, FBoV-2, and FBoV-3) have been described worldwide in feline populations [16,19]. FBoV-1, FBoV-2 and FBoV-3 have been reclassified as carnivore bocaparvovirus-3, carnivore bocaparvovirus-4 and carnivore bocaparvovirus-5, respectively [24].

The pathogenic role of FBoV is unclear as FBoV has been identified in both healthy cats and cats with clinical disease [10]. Regarding the potential pathogenicity, similarly to other bocaviruses, FBoV has been identified in cats presenting with diarrhea, including hemorrhagic enteritis [13,16,17,23]. Nervous system infection associated with FBoV infection has been observed in cats [20] and recently FBoV has also been considered a potentially neurotropic virus [3,14,18].

The epidemiology, evolution, diversity and pathogenicity of FBoV in cats are still under investigation. Mirroring bocavirus infections in other animals, further studies are still required to better understand the potential role of FBoV also in respiratory and urinary tract disorders in cats.

Due to the limited number of studies on FBoV in Italy, the aims of this observational study based primarily on PCR detection were (i) to investigate the presence of FBoV viral DNA in cats from Italy using PCR methods and sequencing analysis to characterize circulating FBoV strains, (ii) to investigate the potential pathogenic role of FBoV by investigating FBoV viral DNA in fecal, urine, respiratory and blood samples from cats and (iii) to assess the association between FBoV positivity, clinical status and patient variables.

## 2. Materials and Methods

### 2.1. Animals and Sampling

This observational study investigated FBoV occurrence by convenience sampling of 224 cats admitted to the Veterinary Teaching Hospital of Lodi, Italy for routine diagnostic procedures between 2020 and 2025. Blood, feces, urine, respiratory specimens or other types of samples were collected from cats.

All samples were collected in the context of clinicopathological investigations or as part of health checks requested by the referring veterinarians following informed consent of the owners. Following diagnostic procedures, samples and/or extracted DNA were subsequently stored at −80 °C for further molecular analysis.

All samples were collected for diagnostic purposes or as part of health checks and with informed consent of the owners and therefore according to the guidelines of the Institutional Animal Care and Use Committee of the University of Milan, a formal approval was not required.

Data regarding time of sampling, sex, age, and type of ownership were recorded for each animal. Age was categorized as juvenile (<1 year) and adult (≥1 year) [14]. Data from routine diagnostic analysis based on qPCR to detect Feline panleukopenia virus (FPV) in fecal samples, according to a published protocol [25], were also recorded. For fecal samples only, the occurrence of diarrhea and FPV positivity was also considered.

### 2.2. FBoV Detection by PCR

DNA was extracted from whole blood, respiratory and urine samples using a commercial DNA extraction kit (NucleoSpin Blood^®^ kit, Macherey-Nagel, Düren, Germany), following manufacturer’s instructions. DNA was extracted from fecal samples using a commercial DNA extraction kit (NucleoSpin^®^ Tissue Kit, Macherey-Nagel, Düren, Germany), according to the manufacturer’s instructions. The extracted DNA was stored at −20 °C until further molecular analysis. DNA pre-analytical quality control was performed on all samples by a PCR targeting vertebrate 12S rRNA locus [26].

A PCR capable of simultaneously detecting FBoV-1, FBoV-2, and FBoV-3 by targeting a proportion of the NP1 gene was performed as previously described. The PCR amplifies 464, 503 and 491 bp fragments for FBoV-1, FBoV-2 and FBoV-3, respectively [27]. The PCR assay was not analytically validated for sensitivity and specificity in the present laboratory. PCR was performed in a final reaction volume of 25 μL containing 1x Phire Green Hot Start II PCR (Thermo Fisher Scientific, Segrate –Milan, Italy), 1 μM of forward primer FBoV1-2-3 NP1 F (5′- AGA ACC RCC RAT CAC ART CCACT-3′), 1 μM reverse primer FBoV1-2-3 NP1 R (5′- TGG CRA CCG CYA GCA TTT CA-3′), and 5 μL of template DNA. Amplification was performed with an initial denaturation step at 98 °C for 30 s, followed by 40 cycles of denaturation at 98 °C for 5 s, annealing at 51 °C for 5 s, and extension at 72 °C for 10 s. A final extension step was performed at 72 °C for 1 min. PCR products were separated by electrophoresis on 1.5% agarose gel and visualized under UV light. A positive control (a FBoV field sample previously obtained in our laboratory and confirmed by sequencing as FBoV-3) and a blank control (DNAse-free water sample) were also included in the PCR reaction.

When multiple samples were collected and analyzed from the same animal, cats were considered FBoV-positive if at least one sample was positive.

Samples showing a band of the expected size were further subjected to sequencing for characterization of FBoV genotype.

### 2.3. FBoV Genotype and Phylogeny

Amplicons from PCR-positive results were purified and sequenced using the same internal forward and internal reverse primers used in the DNA amplification. Sanger sequencing was performed by a commercial sequencing facility (Microsynth Seqlab, Germany). BioEdit software version 7.0 https://thalljiscience.github.io/ (accessed on 3 March 2026) was used to assemble the sequence data. BLAST https://blast.ncbi.nlm.nih.gov/Blast.cgi (accessed on 10 July 2026) was used to compare the sequences with those available in GenBank. Clustal X in BioEdit software v.7.0 was used to align all the consensus sequences against the FBoV genotypes retrieved from GenBank to identify the FBoV genotype of the strain.

Sequences aligned with FBoV reference sequences retrieved from GenBank were subjected to phylogenetic analysis using Clustal X in BioEdit software v.7.0. In the presence of identical sequences from the same animal, only one representative sequence/cat was selected and phylogenetically analyzed. Maximum likelihood (ML) methods with 1000 bootstrap replicates using MEGA v.12 were used for phylogeny [28,29,30]. The General Time Reversible model with Gamma distribution and invariant sites used for phylogeny based on the ML method was selected according to the lowest AIC value.

For sequence comparison, the percentage of nucleotide similarity of pairwise evolutionary distances was calculated using MEGA v.12.

The sequences generated in this study were submitted to GenBank with accession numbers PZ787303- PZ787319.

### 2.4. Hematological and Biochemical Tests

In order to investigate the presence of hematological or biochemical changes possibly associated with FBoV positivity, the database of the VTH was examined to assess whether cats that were PCR positive also underwent hematological or biochemical analyses.

When requested by the clinical presentation, blood was collected on the same day as the collection of samples submitted for PCR. More specifically, 1 mL of blood was placed in EDTA tubes, and the remaining amount was placed in tubes without anticoagulant. Tubes were immediately submitted to the clinical pathology laboratory of the VTH. EDTA tubes were used to perform a complete blood cell count (CBC) with the laser-based analyzer Sysmex XN1000V (Sysmex Corporation, Kobe, Japan). Tubes without anticoagulant were centrifuged (10 min, 4500 rpm) and serum was used to perform a routine clinical chemistry panel using the spectrophotometer BT3500 (Futurlab Srl, Limena PD, Italy).

### 2.5. Data Analysis

For FBoV detection in cats, a sample size of 224 animals was calculated using WinEpi (http://www.winepi.net/uk/index.htm, accessed on 1 October 2025), considering a population size of approximately 6000 cats admitted to the VTH in 2020–2025 for routine diagnostic analysis, a minimum prevalence of 1.3% calculated as the mean positivity in cat’s samples with low positivity [10], and 95% confidence level.

Pearson’s chi-square test or Fisher’s exact test was used to evaluate the differences between proportions of FBoV-positive animals and sex, age categories and hospitalization. In the subgroup of cats with analyzed fecal samples, Fisher’s exact test was also used to evaluate the differences between proportions of FBoV-positive animals and parvovirus positivity and presence of diarrhea. In the presence of more than one fecal sample from the same cat, a cat was considered FPV-positive if at least one sample was FPV-positive. Statistical comparisons based on Pearson’s chi-square test or Fisher’s exact test and calculation of odds ratio (OR) with 95% confidence intervals (CI) were carried out using Epitools [31], taking *p* < 0.05 as significant. The statistical unit used for each analysis was represented by the animal.

Sample-level data were also reported for detection of FBoV and FPV single positivity or co-positivity in samples from cats with and without diarrhea as purely descriptive results, and these data were not statistically analyzed.

## 3. Results

### 3.1. Animals and Samples Tested

Out of the 224 total cats tested for FBoV, the median age was five years and two months, ranging from 1 month and 16 days to 18 years of age. Data of the animals’ characteristics based on number of cats analyzed, including presence of diarrhea and FPV positivity in cats from which fecal samples were collected are reported in Table 1.

A total of 263 samples were collected from these 224 cats. The samples comprised 80 fecal, 76 blood, 54 respiratory, 51 urine, and two other types of samples, collected from 53, 69, 54, 46 and two cats, respectively. The number of samples collected from each animal varied, and one sample was collected from 201 (89.7%) cats, whereas more than one fecal, blood or urine sample was collected from 23 (10.3%) cats. Regarding the subgroup of 53 cats with analyzed fecal samples, one fecal sample was collected from 39 (73.6%) cats, and more than one sample was collected from 14 (26.4%) cats, ranging from two to six samples (Table 2).

### 3.2. FBoV Occurrence and Genotype

In this observational study, FBoV was detected by PCR and confirmed by sequencing in 17/224 cats (7.6%; 95% C.I: 4.1–11.1%), corresponding to 19/263 (7.2%) positive samples. Results of FBoV-positive cats are reported in Table 1.

Among the 53 cats with analyzed fecal samples, when considering the 39 cats with only one fecal sample collected, results showed FBoV positivity in 10 (25.6%) cats. For the remaining 14 cats that underwent multiple fecal sampling, the results of FBoV PCR in each sample are reported in Table 2. Results in Table 2 show that out of the seven (50%) FBoV-positive cats, FBoV was detected in one sample only in five (71.4%) cats, and in two samples in two (28.6%) cats. The interval between collection of these two positive samples was one week in one cat (cat n. 36) and two weeks in the other cat (cat n. 78). All the seven FBoV-positive cats with multiple analyzed fecal samples were hospitalized for the whole period of sample collection.

Sequencing aimed at FBoV genotype identification was successfully obtained for all the FBoV-PCR positive samples. BLAST analysis of the NP1 fragments of the sequences from this study showed highest nucleotide identity ranging from 96.4% to 99.4% with FBoV sequences deposited in GenBank. For 10 sequences from this study, the highest nucleotide similarity was observed with FBoV sequences recently reported in Italy [23]. Moreover, four sequences from this study showed the highest nucleotide homology with a sequence from the USA (GenBank accession KM017745), two with a FBoV sequence from China (GenBank accession n. OR544348) and one with a FBoV sequence from Thailand (GenBank accession n. PP592986).

Nucleotide identity among the sequences from this study ranged from 55.2% to 100%. Identical FBoV sequences obtained from two positive samples collected from the same cats (cat n. 36 and cat n. 78) were observed. Among sequences from different cats, 100% nucleotide identity was observed in three (cats 214, 218, 219), two (cats 60, 62) and in two sequences (cats 63, 66).

According to sequencing results and BLAST analysis, FBoV-1, FBoV-2 and FBoV-3 were detected in nine (4%), two (0.9%) and six (2.7%) cats, respectively. Of the 17 positive cats, more than half (nine, 52.9%) harbored FBoV-1, followed by FBoV-3 in six (35.3%) and FBoV-2 in two (11.8%) cats.

**Figure 1:**
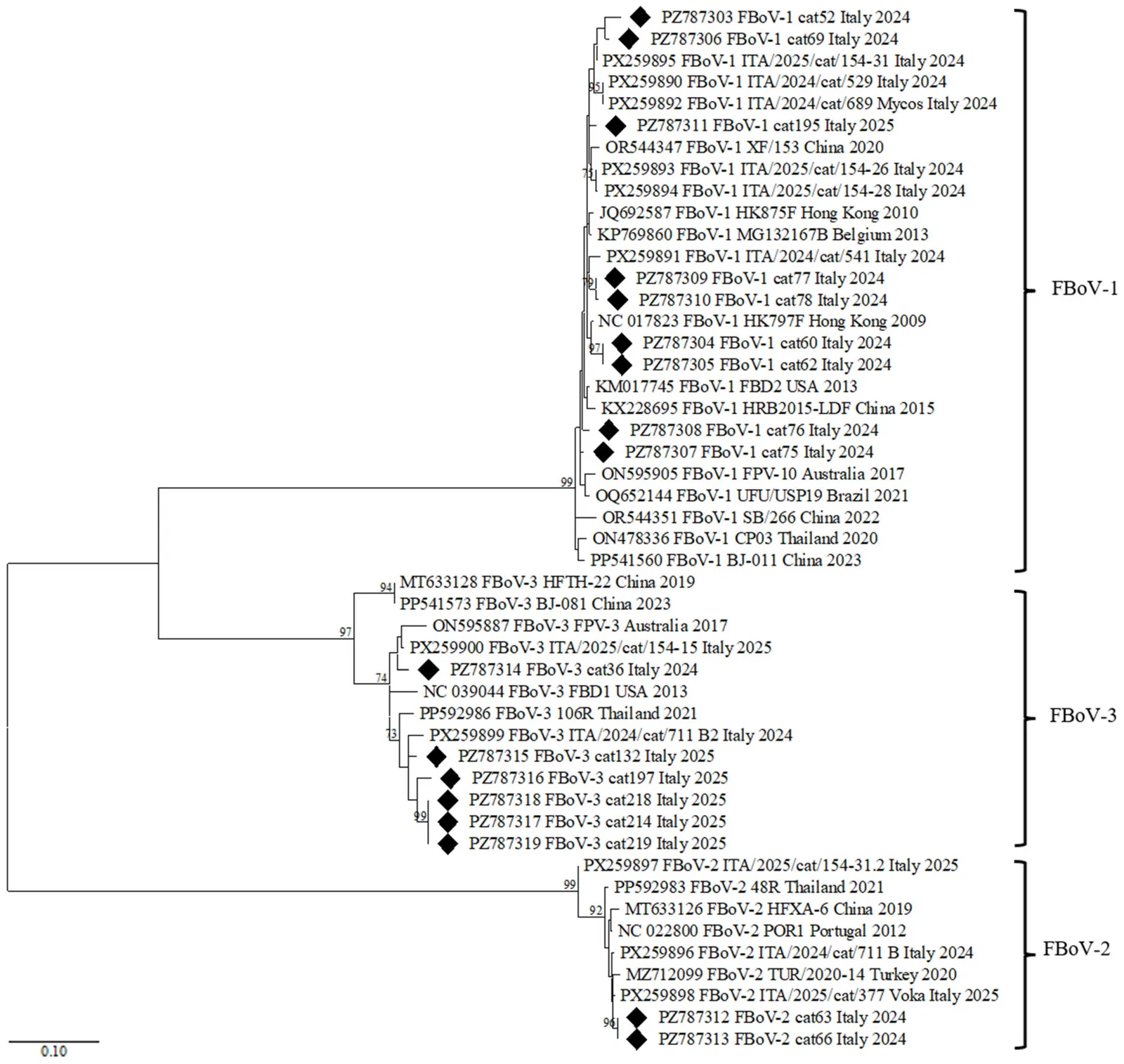
Phylogenetic tree generated with ML analysis inferred from 48 sequences. A 465–491 bp region of the NP1 gene obtained from FBoV sequences retrieved from GenBank databases and the 17 FBoV sequences obtained in this study was used. One sequence per cat from this study was included when identical sequences were obtained from multiple samples. Reference sequences for FBoV-1, FBoV-2 and FBoV-3 are represented by GenBank accession numbers NC_017823, NC_022800 and NC_039044, respectively. Sequences are indicated by GenBank accession number https://www.ncbi.nlm.nih.gov/genbank/ (accessed on 10 July 2026), FBoV genotype, strain identification, country, and year of collection. Distances were computed using the General Time Reversible model with Gamma distributed and invariant sites. Bootstrap values above 70% are given. The sequences obtained in this study are shown with black diamonds.

Results of phylogeny based on ML analysis of FBoV sequences detected in this work (one sequence/cat) are reported in Figure 1. Phylogeny confirmed that the sequences obtained from cats in this study clustered with either FBoV-1, FBoV-2 or FBoV-3 sequences retrieved from GenBank.

Phylogeny showed the presence of significant subclades with bootstrap values > 90% among some of the sequences from this study corresponding to sequences with 100% nucleotide identity. These sequences were obtained from three FBoV-3 sequences (cats n. 214, n. 218 and n. 219), two FBoV-1 sequences (cats n. 60 and 62) and two FBoV-2 sequences (cats n. 63 and n. 66) detected in cats that were either from the same household (cats n. 218 and n. 219) or hospitalized during the same days. Samples from cats with identical FBoV-1 and FBoV-2 sequences were part of the same extraction/PCR batch including other samples with either negative results or FBoV positivity with different FBoV genotypes and sequences. Samples from cats with identical FBoV-3 sequences were also part of the same extraction/PCR batch including other negative samples.

The results of single positivity of FBoV or FPV and co-positivity of FBoV genotypes and FPV according to diarrhea status in 53 cats with fecal samples collected were discordant in some of the cats with multiple samplings. In these animals, different samples from the same cat showed single positive results for FBoV in one sample and FPV in other samples, or single positive results for FBoV or FPV in one sample and simultaneous detection of FBoV and FPV in other samples (Supplementary Table S1).

Therefore, the descriptive results regarding the detection of single positivity or co-positivity for FBoV genotypes and FPV are reported according to individual fecal samples (Table 3). Results show that, among FBoV-positive samples from cats with diarrhea, FBoV-1 and FPV co-positivity accounted for the highest percentage of positive samples.

### 3.3. Variables Associated with FBoV Positivity

Results of statistical analysis based on Pearson’s chi-square test or Fisher’s exact tests to evaluate differences in the proportions of FBoV-positive cats according to the variables are reported in Table 1. Young age, stray/shelter status, hospitalization and FPV positivity in cats with fecal samples analyzed were significantly associated with FBoV positivity. No correlation was detected between FBoV positivity and the other variables considered.

No evident association with clinicopathological changes was found; hematological and biochemical data were available for 12 of the 17 positive cats. Clinicopathological testing was almost negligible for 3/12 cats: two of these were slightly anemic and one had increased urea and hematocrit potentially consistent with dehydration. The remaining 9/12 cats presented with hyperglycemia, which was associated with panleukopenia in 5 cases, and with lymphopenia and neutrophilic leukocytosis with toxic changes in 3 cases. In most cases these changes were associated with clinical signs, FPV or FIV or FeLV positivity (Table 4).

## 4. Discussion

Feline bocavirus (FBoV) is classified into three distinct genotypes and is currently considered one of the emerging viral agents affecting cats, although its pathogenic potential remains unclear. This study investigated FBoV in Northern Italy, demonstrating that FBoV was detected among cats examined at the Veterinary Teaching Hospital of Lodi, showing that the virus is not only circulating in Southern Italy, as recently reported [23].

The 7.6% overall positivity observed by PCR analysis in the study population of cats by convenience sampling, corresponding to 7.2% of positive samples, was based on analysis of fecal, respiratory, urine and blood samples and may have been influenced by the sampling method used in this study. Indeed, animals were recruited for diagnostic purposes, and convenience sampling does not allow us to infer FBoV prevalence in the Italian cat population. A possible overestimation bias due to this type of sampling may have occurred. However, the overall positivity detected in our study is in line with the 2.5% positivity observed in different types of samples in China collected from 364 cats [10].

Depending on the specific sample type, the detection of FBoV in fecal samples in our study was expected and has been reported previously. The 32% positivity observed in the cats with fecal samples analyzed of this work agrees with studies showing prevalence of 25.9% in cats’ fecal samples from China [2] and 22.2% in rectal swabs collected from Italian cats with acute gastroenteritis [23]. However, lower prevalence than that detected in our study has also been reported, with positivity ranging from 2.4% to 9.9% in rectal swabs/fecal samples from cats in China, Vietnam and Japan [10,16,19]. In addition to the potential overestimation related to convenience sampling, variations in positivity among different studies may be due to several factors, including differences in cat populations, geographical areas, and diagnostic approaches used to detect FBoV. Indeed, the PCR assay used in this study was not analytically validated for sensitivity and specificity in the present laboratory and therefore false positive and false negative results cannot be ruled out, which may have affected the estimated FBoV frequency in this study.

The FBoV-negative results in cats with blood, respiratory and urine samples analyzed indicate that FBoV was not detected in the relatively limited number of samples examined using the methodology employed. Therefore, low-level or intermittent detection in these sample types cannot be excluded and further studies involving a large number of samples are required to confirm these results. Indeed, while a very low prevalence has been reported in blood, respiratory and urine samples from cats [10], a few studies have observed higher positivity [14,22,32].

Our results showing the detection of all three FBoV genotypes in cats from Italy, with FBoV-1 being the most frequently detected, confirm the circulation of all three FBoV genotypes in cat populations recently reported in Italy and previously reported in other countries. FBoV-1 was also the most frequently detected FBoV genotype in our study, in agreement with previous findings [23,33]. The detection of identical FBoV sequences in consecutively numbered cat samples was verified. Based on the laboratory processing and the results obtained, potential batch-related artifacts were excluded, except for two cats from the same household that were processed consecutively on the same day.

The detection of different strains from different cats in the majority of animals from this study supports the hypothesis of inter-host genetic diversity previously reported [10]. However, the use of a single NP1 target may have affected the detection of genetically divergent strains and the sequences identified in this study may not comprehensively represent the genetic diversity of FBoV circulating in Italy.

Young age and stray/shelter status as variables associated with FBoV positivity have previously been reported as risk factors for FBoV infection [2,4,32,33], although other studies did not identify age or ownership status as associated with FBoV infection [16,19]. FBoV positivity in young cats may be explained by their immature immune system whereas stray/shelter cats may be at higher risk of FBoV positivity compared to owned cats because of the crowded living conditions and constant exposure to other infected cats, suggesting that a high-stress environment may be an important factor contributing to the circulation of FBoV [2]. However, as stress was not measured in this study, additional investigations are needed to confirm this aspect.

Regarding the pathogenicity of FBoV, it should be noted that this was an observational study based primarily on PCR detection rather than a study designed to establish causality. Nevertheless, the finding of FBoV exclusively in fecal samples may suggest a potential pathogenic role in gastrointestinal disease. Epidemiological investigations in different countries have suggested that FBoV may cause diarrhea in cats with a prevalence of 5.5–25.9% [2,10,15,16,23] and may also be associated with outbreaks of hemorrhagic enteritis [17]. However, our study did not confirm the overall association between FBoV infection and diarrhea that was previously reported in cats from China [2,27]. On the other hand, our results are in line with previous studies showing similar FBoV positivity in fecal samples collected from cats with and without gastrointestinal symptoms [19], suggesting that FBoV may not be a primary pathogen to cause diarrhea in cats. Moreover, hematological or biochemical data, when present, were nonspecific (e.g., hyperglycemia not associated with other hematological or biochemical changes, likely induced by stress) or associated with inflammatory intestinal disease, particularly in cats with parvoviral positivity and panleukopenia. These findings suggest that the possible FBoV positivity does not induce specific hematological or biochemical changes other than those induced by the primary intestinal disease, when present. However, hematological and biochemical results should be interpreted cautiously because only 12 of the 17 positive cats had complete clinicopathological data and the absence of specific abnormalities may not exclude clinically relevant effects of FBoV. Furthermore, these data were obtained according to clinical indications rather than under a standardized prospective protocol. Therefore, additional studies are needed to evaluate systematically whether FBoV infection is associated with specific hematological or biochemical alterations.

Epidemiological investigations have shown that coinfections with FPV and FBoV are common [23,34]. FPV and FBoV coinfections have been identified with higher frequency in cats with diarrhea, whereas healthy cats show higher frequency of monoinfections [27]. The detection of FBoV and FPV single positivity or co-positivity observed in our study in samples from cats with diarrhea require further investigations to confirm our findings. Indeed, the results from this study were purely descriptive, were obtained from very small numbers of individual fecal samples and some observations were based on multiple samples from the same cat sometimes showing discordant results in different samples. Therefore, the observations in this fecal-sample analysis cannot be considered independent and no inferential conclusions can be drawn. Moreover, the limited numbers of samples from cats with diarrhea did not allow us to understand if FBoV-1 has greater pathogenicity than the other FBoV genotypes. Other studies have found FBoV-1 and FBoV-2 to be significantly more associated with diarrhea in kittens than FBoV-3 [33]. The different pathogenicity of FBoV types requires further investigation to determine whether these differences may instead be due to the higher frequency of FBoV-1 detection, given that our study also showed that FBoV-1 is the only FBoV genotype detected with single positivity in cats. Indeed, the higher detection of FBoV-1 in cats with diarrhea may reflect differences in genotype frequency rather than differences in virulence or pathogenicity.

The observed association between FBoV positivity and hospitalization was not surprising. The negative results at the first sampling followed by positivity at the 2nd or 3rd sampling in some of the hospitalized cats with multiple samples warrant further prospective investigation to define if FBoV positivity may occur in cats after admission to veterinary hospitals. The detection of the same FBoV sequences in some cats hospitalized on the same days further requires additional study to investigate potential transmission between cats during hospitalization. Indeed, the present observational design did not investigate FBoV prior to admission to determine whether positivity occurred after admission in all cats. It is also not possible to rule out the possibility that cats were hospitalized because of clinical conditions that themselves increased the likelihood of FBoV detection or because they were more likely to undergo fecal sampling. In addition, important potential confounding factors, including age, ownership status, clinical disease, sampling type, and FPV infection, may be associated with hospitalization. Therefore, the association between FBoV positivity and hospitalization observed in this study should be interpreted with caution, and further prospective studies are needed to investigate the possibility of nosocomial transmission. Different nosocomial infections, also referred to as healthcare-associated infections (HAIs), have been reported in veterinary hospitals and clinics [35]. Up to now, attention has focused on bacterial infections, especially those caused by antimicrobial resistant bacteria, but nosocomial infections can also be caused by non-bacterial pathogens, including viruses, fungi, and parasites [36]. In veterinary settings, FPV has emerged as one of the viruses with the highest risk of nosocomial transmission, and viruses are of particular concern because parvoviruses are characterized by high environmental stability, ease of transmission, and often rapid spread within hospital populations [37]. Considering that FBoV, like FPV, belongs to the family *Parvoviridae*, and that environmental contamination by FBoV has recently been reported [38], the risk of FBoV transmission in veterinary hospitals requires further investigation.

FBoV transmission among cats is likely via the fecal route [13,39]. Although this study did not focus on the duration of FBoV fecal shedding, our preliminary results on a limited number of cats with more than one fecal sample collected suggest that FBoV-positivity generally lasts for a few days, whereas a minority of cats may have prolonged fecal shedding lasting for at least two weeks. Similarly, prolonged FBoV viral shedding after clinical recovery has been recently reported, and in one kitten FBoV DNA has been detected up to 65 days [38]. This aligns with the shedding duration reported for other viruses belonging to the *Parvoviridae* family, such as FPV in cats, for which shedding typically lasts 1–2 or 5–7 days, and rarely up to 6 weeks as reported in one cat in an early study [40]. However, further studies are needed to confirm the duration of FBoV fecal shedding in cats as well as the presence (and duration) of environmental contamination, to further understand the risk of transmission.

This study has some limitations. First of all, this was an observational study and was not designed to establish causality of FBoV in diseased cats. Given the limited number of FBoV-positive cats, statistical analysis based on a multivariable model was not performed. Therefore, the identified association between variables and FBoV-positivity could not be confirmed as independent risk factors. Moreover, the cause of disease was not determined, as other feline diarrhea-, respiratory- or CKD-associated pathogens were not investigated. Our study did not investigate the presence of FBoV in different samples from the same animal to confirm wide tissue distribution previously reported [10]. In this study, the use of only a fragment of the NP1 gene for FBoV genotype identification may have affected the detection of genetic differences among strains. Therefore, the genetic diversity of the FBoV strains detected in this study may have been underestimated. Further studies based on whole genome sequencing are mandatory to fully characterize the genetic features of FBoV strains circulating in Italy.

## 5. Conclusions

Results of this observational study showing the detection of FBoV-1, FBoV-2 and FBoV-3 in cats from Italy need to be interpreted with caution as the convenience sampling limits the generalizability of the observed FBoV positivity to the wider Italian cat population. This study did not establish a causal role of FBoV in gastrointestinal disease. Nevertheless, the detection of FBoV highlights the need for further studies on the genetic features, epidemiology, shedding dynamics and pathogenicity in FBoV-positive cats from Italy.

## Figures and Tables

**Table 1:** Number and percentage of FBoV-positive animals according to characteristics of 224 cats.

Variable	Category	No. Tested	No. FBoV-Positive (%)	Odds Ratio (95% CI)	*p* Value *
Sex **	female	87	9 (10.3)	1.83 (0.68–4.95)	0.3014
	male	135	8 (5.9)		
Age **	juvenile (<1 year)	45	9 (20)	6.62 (2.22–19.8)	**0.0008**
	adult (≥1 year)	165	6 (3.6)		
Type of ownership **	stray/shelter	76	11 (14.5)	3.95 (1.4–11.14)	**0.0139**
	privately owned	146	6 (4.1)		
Sample	fecal	53	17 (32.1)	nd	nd
	blood	69	-		
	respiratory	54	-		
	urine	46	-		
	other	2	-		
Cats with fecal sample analysis: presence of diarrhea in cats **	yes	20	8 (40)	1.92 (0.58–6.38)	0.36
	no	31	8 (25.8)		
Cats with fecal sample analysis: Parvovirus positivity in cats	yes	29	16 (55.2)	28.31 (3.36–238.6)	**<0.0001**
	no	24	1 (4.2)		
Hospitalization **	yes	96	15 (16.6)	12.96 (1.67–100.6)	**0.0023**
	no	71	1 (1.4)		

CI: confidence intervals; nd = not performed. * *p* values based on Pearson’s chi-square test or Fisher’s exact test results to evaluate the differences between proportions of FBoV-positive animals and variables. ** Sex was not available for two cats; age was not available for 14 cats; type of ownership was not available for two cats. Data on the presence of diarrhea was not available for two cats; hospitalization status was unknown for 57 cats.

**Table 2:** FBoV PCR results for each fecal sample collected from a subset of 14 cats with multiple fecal samples analyzed.

Cat ID	No. of Fecal Samples Analyzed	FBoV Result in Cat	FBoV Result for Each Sample Analyzed					
			1st	2nd	3rd	4th	5th	6th
28	2	neg	-	-				
52	2	pos	-	+				
56	2	neg	-	-				
60	2	pos	-	+				
78	2	pos	+	+				
132	2	pos	+	-				
160	2	neg	-	-				
186	2	neg	-	-				
187	2	neg	-	-				
72	3	neg	-	-	-			
63	4	pos	-	-	+	-		
37	5	neg	-	-	-	-	-	
214	5	pos	-	+	-	-	-	
36	6	pos	+	+	-	-	-	-

blank = sample not collected.

**Table 3:** Descriptive results of FBoV and FPV single positivity or co-positivity in a total of 80 individual fecal samples collected from 53 cats and analyzed in this study, according to diarrhea status.

	No. of Samples	No. (%) Single Positivity			No. (%) Co-Positivity			
		Total Single Positivity	FBoV-1	FPV	Total Co-Positivity	FBoV-1 + FPV	FBoV-2 + FPV	FBoV-3 + FPV
Presence of diarrhea	21	7 (33)	1 (5)	6 (29)	5 (24)	4 (19)	-	1 (5)
Absence of diarrhea	57	25 (44)	3 (5)	22 (39)	9 (16)	2 (4)	2 (4)	5 (9)
Total *	80	32 (40)	4 (5)	28 (35)	15 (18.8)	6 (8)	2 (3)	7 (9)

* including two samples (from two cats) for which data on diarrhea status were not available.

**Table 4:** Results of blood analyses in the 12 FBoV-positive cats that underwent complete clinicopathological testing, and association with FPV positivity and presence or absence of diarrhea.

FBoV	FPV	Diarrhea	Results of Blood Testing
FBoV-3	pos	No	Hyperglycemia, panleucopenia
FBoV-1	neg	Yes	Hyperglycemia, inflammatory leukogram *
FBoV-1	neg	No	Slight anemia **
FBoV-2	pos	No	Hyperglycemia, panleucopenia
FBoV-2	pos	No	Increased Ht and urea
FBoV-1	neg	No	Slight anemia *
FBoV-1	pos	Yes	Hyperglycemia
FBoV-1	pos	Yes	Hyperglycemia, inflammatory leukogram
FBoV-3	pos	No	Hyperglycemia, panleucopenia
FBoV-1	pos	No	Hyperglycemia, inflammatory leukogram
FBoV-3	pos	Yes	Hyperglycemia, panleucopenia
FBoV-3	pos	No	Hyperglycemia, panleucopenia

* FIV-positive; ** FeLV-positive.

## Data Availability

The data that supported the findings of this study are available from the corresponding author upon reasonable request.

## Supplementary Materials

The following supporting information can be downloaded at https://www.mdpi.com/article/10.3390/pathogens15101040/s1, Table S1: FBoV and FPV PCR results in multiple samples collected from 14 cats and analyzed in this study.

## Abbreviations

The following abbreviations are used in this manuscript:

AIC	Akaike Information Criterion
BLAST	Basic Local Alignment Search Tool
BoVs	Bocaparvoviruses
CBC	Complete Blood Count
CKD	Chronic Kidney Disease
DNA	Deoxyribonucleic Acid
EDTA	Ethylenediaminetetraacetic Acid
FBoV	Feline Bocavirus
FBoV-1	Feline Bocavirus genotype 1
FBoV-2	Feline Bocavirus genotype 2
FBoV-3	Feline Bocavirus genotype 3
FPV	Feline Panleukopenia Virus
HAIs	Healthcare-Associated Infections
HBoV	Human Bocavirus
ICTV	International Committee on Taxonomy of Viruses
IRIS	International Renal Interest Society
ML	Maximum Likelihood
NP1	Non-Structural Protein 1
PCR	Polymerase Chain Reaction
qPCR	Quantitative Polymerase Chain Reaction (Real-Time PCR)
rRNA	Ribosomal Ribonucleic Acid
SG	Specific Gravity
uGGT	Urinary Gamma-Glutamyl Transferase
UPC	Urine Protein-to-Creatinine Ratio
USA	United States of America
VTH	Veterinary Teaching Hospital
