# Supplementary material for: Genotypic Diversity of Feline Bocavirus Identified in Cats from Italy
Source: Pathogens. 2026 Oct 1;15(10):1040. doi: 10.3390/pathogens15101040 (PMC13708229; doi:10.3390/pathogens15101040)
Supplement: Supplementary file 1 [file pathogens-15-01040-s001.zip › pathogens-4515457-supplementary.pdf]

Supplementary Table S1. FBoV and FPV PCR results in multiple samples collected from 14 cats and analyzed in this study.

Cat ID	No. of Analyzed Fecal Samples	FBoV-Result in Cat	FBoV and FPV PCR Result in Each Analyzed Sample					
			1st	2nd	3rd	4th	5th	6th
28	2	neg	FPV	neg				
52	2	pos	FPV	FBoV-1				
56	2	neg	FPV	FPV				
60	2	pos	FPV	FBoV1				
78	2	pos	FBoV-1/FPV	FBoV-1				
132	2	pos	FBoV-3/FPV	neg				
160	2	neg	FPV	neg				
186	2	neg	FPV	neg				
187	2	neg	FPV	neg				
72	3	neg	FPV	FPV	neg			
63	4	pos	FPV	FPV	FBoV-2/FPV	neg		
37	5	neg	FPV	FPV	FPV	FPV	neg	
214	5	pos	FPV	FBoV-3/FPV	FPV	FPV	neg	
36	6	pos	FBoV-3/FPV	FBoV-3/FPV	FPV	FPV	FPV	neg

blank = sample not collected
